# Folic Acid in the Treatment of Sickle Cell Disease: A Systematic Review

**DOI:** 10.7759/cureus.57962

**Published:** 2024-04-10

**Authors:** Divine Besong Arrey Agbor, Priyanka Panday, Samrah Ejaz, Simhachalam Gurugubelli, Suviksh K Prathi, Yaneisi Palou Martinez, Sondos T Nassar

**Affiliations:** 1 Clinical Research and Internal Medicine, California Institute of Behavioral Neurosciences & Psychology, Fairfield, USA; 2 Internal Medicine, Richmond University Medical Center, Staten Island, USA; 3 Research, California Institute of Behavioral Neurosciences & Psychology, Fairfield, USA; 4 Internal Medicine, California Institute of Behavioral Neurosciences & Psychology, Fairfield, USA; 5 Internal Medicine, Memorial Health System, Gulfport, USA; 6 Medical School, California Institute of Behavioral Neurosciences & Psychology, Fairfield, USA; 7 Medical School, St. George's University School of Medicine, St. George's, GRD; 8 Medicine, California Institute of Behavioral Neurosciences & Psychology, Fairfield, USA; 9 Medicine and Surgery, Jordan University of Science and Technology, Irbid, JOR

**Keywords:** vitamin b9, sickle cell anemia, sickle cell disease, folate, folic acid

## Abstract

Sickle cell disease (SCD) is a group of inherited genetic disorders that is caused by a mutation in the gene that codes for hemoglobin subunit β. This systematic review aimed to evaluate the effect of folic acid in the treatment of SCD patients. We retrieved 3730 articles from PubMed, PubMed Central, Google Scholar, and ScienceDirect databases. We employed a search technique that involved framing keywords, such as folic acid, folate, and sickle cell illness, and the Medical Subject Headings (MeSH) strategy in PubMed. We chose research articles that had been published during the last 10 years, as well as case reports, systematic reviews and meta-analyses, literature reviews, randomized controlled trials, and observational studies. Exclusion criteria included paid full-text articles, abstracts, non-English studies, and patients who do not have SCD. The 2020 Preferred Reporting Items for Systematic Reviews and Meta-Analyses (PRISMA) criteria were used in the design of our systematic review. It was found that the majority of SCD patients were receiving regular folic acid supplements and that their plasma folate levels were either increased or within normal range, with no discernible impact on other clinical outcomes such as hemoglobin levels, infections, or pain crises. SCD patients produce more red blood cells than healthy individuals, and nearly all SCD patients receive daily folic acid supplements. On the other hand, not enough information is available on folic acid's potential benefits in the management of SCD; thus, there is a need for more large clinical trials.

## Introduction and background

An estimated 250 million people worldwide are thought to be affected with sickle cell disease (SCD), the majority of whom are of African heritage. One in 12 African Americans has the SCD mutation, and one in 500 of them really has the illness. Every year, one in every 16,300 Hispanic-American infants born in the United States is born with sickle cell anemia (SCA) [1,2]. Up to 90% of under five deaths in sub-Saharan Africa can be attributed to it [3], and 500 premature deaths of children with SCD occur every day [4]. This can be the consequence of a delayed diagnosis and/or limited access to all-encompassing care, a tendency that has to be immediately addressed [5]. SCD is a group of inherited genetic disorders that is caused by a mutation in the gene that codes for hemoglobin (Hb) subunit β. The gene mutation causes valine to take the place of glutamic acid at position 6 in the β-globin chain. Erythrocytes with a crescent shape are produced when the polypeptide's main structure changes, converting hemoglobin A to hemoglobin S (HbS) [6,7]. The disease is acquired by inheriting abnormal genes from both parents, the combination giving rise to different forms of SCD. The following are the SCD genotypes: hemoglobin sickle C (HbSC) disease, double heterozygote for HbS and hemoglobin C (HbC) with intermediate clinical severity; HbS/hereditary persistence of fetal Hb (S/HPHP), extremely mild phenotypic or symptom-free; HbSS disease, often known as SCA, homozygote for the Bs globin with a severe or moderately severe phenotype; and HbS/HbE syndrome, a fairly uncommon condition with a generally mild clinical history that is a double heterozygote for HbS and HbE [8]. Complications from SCD often include anemia, infection, acute chest syndrome, stroke, pulmonary hypertension, anaplastic crisis, anemia, and mortality [9]. L-Glutamine, hydroxyurea, voxelotor, blood transfusion, crizanlizumab, hematopoietic stem cell transplant, gene editing treatment (Casgevy™), folic acid, and penicillin are among the current SCD management techniques [10-12]. Given that SCD patients have an increase in erythropoiesis, the majority of studies have shown the use of folic acid as a regular treatment to prevent folic acid deficit and potential megaloblastic anemia in these patients. In people with SCA, the typical half-life of erythrocytes decreases from 120 days to between 15 and 20 days. During sickle cell crises, the half-life is further shortened, which causes the bone marrow to become more active, in order to replace the red blood cells (RBCs) that have been killed. Folate is depleted as a result, increasing the risk of folate insufficiency [13,14]. Nevertheless, there isn't much data to support folic acid use in SCD individuals. The purpose of our study was to evaluate the role that folic acid plays in SCD treatment and to suggest that larger clinical trials be conducted to confirm the benefits of folic acid supplementation for SCD patients.

## Review

Methodology

The Preferred Reporting Items for Systematic Reviews and Meta-Analyses (PRISMA) 2020 guidelines have been followed in the systematic review [15].

Eligibility Criteria

Based on the participants' treatment and outcome components, this review was created. Participants are individuals with crescent-shaped Hb or SCD. Interventions include the use of folic acid, monitoring folate levels, and preventing anemia. Consequently, Table 1 below illustrates more on the inclusion and exclusion standards that were introduced.

**Table 1:** Details of inclusion and exclusion criteria RCT: randomized controlled trial; SCD: sickle cell disease

	Inclusion	Exclusion
Research paper	Folate and sickle cell	Non-folate treatment
Publication date	Last 10 years (2014-2024)	Above 10 years
Literature	Published literature	Author letters, conference abstracts, books, grey literature, non-published literature
Study types and designs	RCT, cohort, systematic reviews, meta-analysis, case reports, observational	
Population	People with SCD	Those not affected by SCD
Sex	Both female and male	
Language	English	
Text availability	Free full text only	Abstracts, paid full text

Database and Search Strategy

Using the databases ScienceDirect, Google Scholar, PubMed, and PubMed Central, a methodical search was carried out. The database was lastly searched in January 2024. Folic acid, folate, sickle cell disease, and crescent-shaped hemoglobin were the terms entered into the search engines, and the Medical Subject Headings (MeSH) approach was employed in PubMed. Table 2 provides more details about PubMed and search techniques.

**Table 2:** Details of search strategy

Databases	Keywords	Search strategy	Filters	Search result
PubMed	Sickle cell anemia, sickle cell, drepanocyte anemia, folates, folic acid, vitamin B9	Folic acid OR Folate OR Vitamin B9 OR ("Folic Acid"[Mesh]) OR ("Folic Acid/administration and dosage"[Majr] OR "Folic Acid/adverse effects"[Majr] OR "Folic Acid/genetics"[Majr] OR "Folic Acid/physiology"[Majr] OR "Folic Acid/supply and distribution"[Majr] OR "Folic Acid/therapeutic use"[Majr] AND Sickle cell disease OR Sickle cell anemia OR Drepanocytic disease OR ("Anemia, Sickle Cell/complications"[Majr] OR "Anemia, Sickle Cell/diet therapy"[Majr] OR "Anemia, Sickle Cell/drug therapy"[Majr] OR "Anemia, Sickle Cell/epidemiology"[Majr] OR "Anemia, Sickle Cell/mortality"[Majr] OR "Anemia, Sickle Cell/pathology"[Majr] OR "Anemia, Sickle Cell/prevention and control"[Majr] OR "Anemia, Sickle Cell/therapy"[Majr] ) 1565	10 years, humans male and female, English	400
Google Scholar	Folic acid, folate, sickle cell, sickle cell anemia	"Folic acid" OR "Folate" AND "Sickle cell anemia" OR "Sickle cell" 18200	Review articles, 10 years	1920
PubMed Central	Folic acid, sickle cell anemia	Folic acid AND sickle cell anemia 1920	10 years	1303
ScienceDirect	Folic acid, sickle cell anemia	Folic acid AND sickle cell anemia 2621	Review articles, research articles, 10 years, open access	107

In EndNote, every reference was sorted and organized alphabetically. We used EndNote and manual effort to eliminate the duplicates. Additionally, the records were filtered by title and abstract, which allowed for the elimination of articles that weren't relevant. The whole text articles were retrieved. In order to reduce the possibility of bias in this study, the articles that were successfully retrieved were evaluated using the proper quality assessment tool.

Quality Assessment

The proper quality rating procedures were employed to assess the quality of the nominated studies. The Joanna Briggs Institute (JBI) critical appraisal methods were used to evaluate cohort studies and cross-sectional research [16]. The scale for the assessment of non-systematic review articles (SANRA) checklist was used to evaluate narrative review evaluations [17]. The systematic reviews and meta-analyses were assessed using the evaluation of the assessment of multiple systematic reviews (AMSTAR) checklist [18]. Finally, we used the Newcastle-Ottawa Scale (NOS) to assess the cohort studies [19]. 

Data Collection Process

After the data was extracted, each co-author contributed equally to the completion of the retrieved data. The core outcomes, such as changes in folic acid levels, and secondary outcomes, such as changes in Hb levels, were thoroughly examined in all of the shortlisted publications. Most of the results indicated how the SCD individual's folate levels were affected.

Results

**Figure 1:**
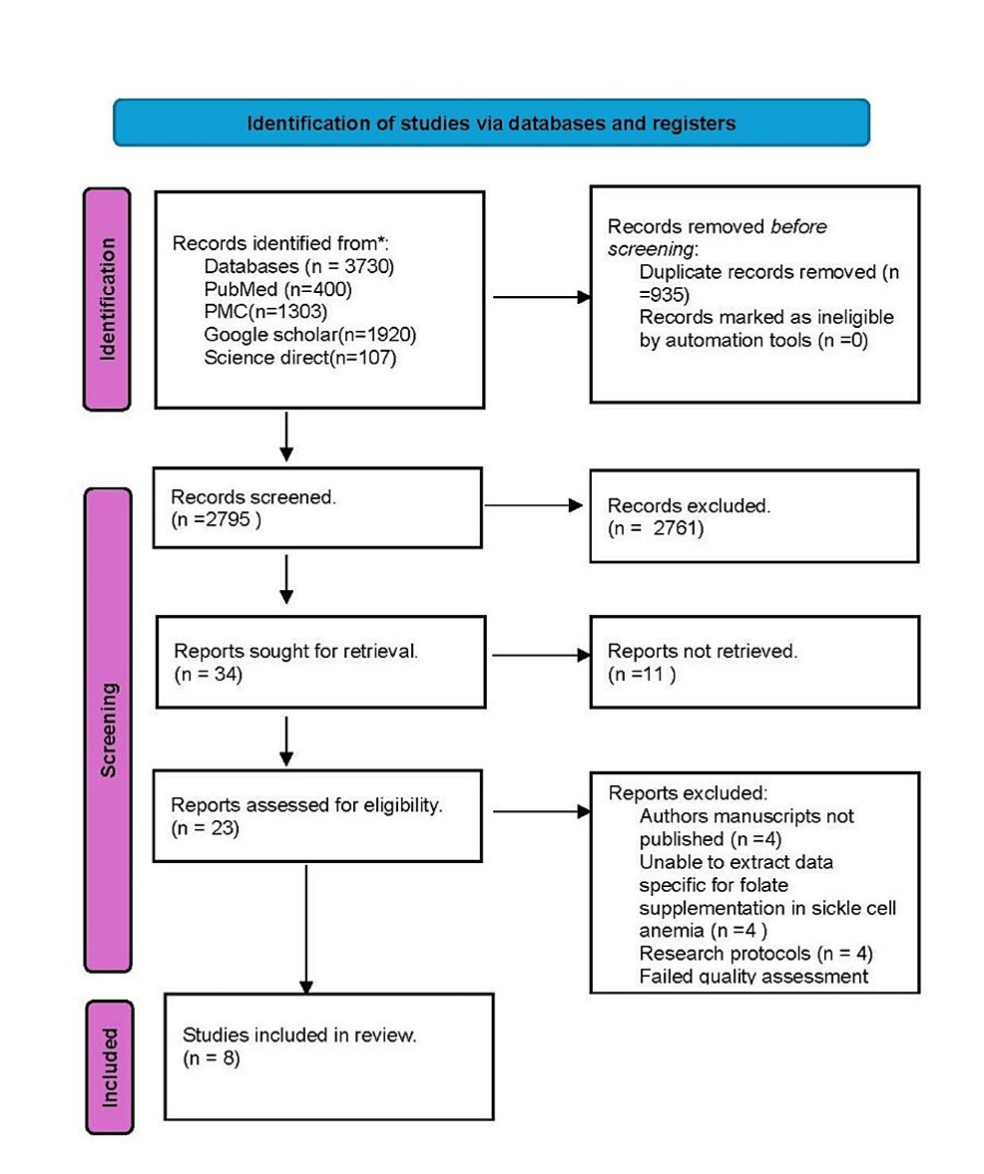
PRISMA flowchart PRISMA: Preferred Reporting Items for Systematic Reviews and Meta-Analyses

We had 3730 articles in our initial search of PubMed Central, PubMed, Google Scholar, and ScienceDirect. EndNote was utilized to eliminate 935 duplicate entries. Following a manual screening process using the titles for the 2795 articles remaining, we discarded 2761 articles. Eleven articles were not retrieved, and 34 were left for retrieval. Using the same eligibility criterion, 23 articles remained. The remaining publications 23 articles were then screened by two different co-authors using full-text, titles, abstracts, and comprehensive inclusion-exclusion criteria. Following careful screening, we included publications that matched our inclusion criteria, which included observational studies, non-randomized controlled trials, and reviews of articles published in English within the last 10 years, as well as any that were pertinent to our study issue. For a comprehensive quality/bias assessment utilizing standardized quality assessment methodologies, a total of 11 papers were included. Following a quality analysis, three papers were deemed unsuitable for inclusion in this systematic review, leaving the remaining eight research studies used. Figure 1 shows the PRISMA 2020 flow diagram [15].

 One systematic review study that was evaluated using the AMSTAR checklist was included in our study [18], as illustrated in Table 3.

**Table 3:** Details of AMSTAR checklist for systematic review article retained in our study To check yes, no, can’t answer, and not applicable. The passing score is >70% AMSTAR: assessment of multiple systematic reviews Reference: [18]

Checklist	Dixit et al. (2018) [20]
Was an "a priori" design provided?	Yes
Were there duplicates in study selection and data extraction?	Yes
Was a comprehensive literature search performed?	Yes
Was the status of publication (e.g., grey literature) used as an inclusion criterion?	Yes
Was a list of studies (included and excluded) provided?	Yes
Were the characteristics of the included studies provided?	Yes
Was the scientific quality of the included study used appropriately in formulating conclusions?	Yes
Were the methods used to combine the findings of studies appropriate?	Yes
Was the likelihood of publication bias assessed?	Yes
Was the conflict of interest included?	Yes

From the quality appraisal carried out, four narrative studies received an acceptable rating from the SANRA checklist [17], as described in Table 4.

**Table 4:** Detailed quality of review articles that met the criteria of the SANRA checklist SANRA 2, Scale for the Assessment of Narrative Review, two articles selected. The passing score is 9/12 SANRA: scale for the assessment of non-systematic review articles Reference: [17]

Publication	Justification of the article	Statement of concrete aims or formulation of questions	Description of the literature search	Referencing	Scientific reasoning	Appropriate presentation of data	Sum	Pass/fail
Hur and Durojaye (2023) [21]	2	1	0	2	2	2	9	Pass
Bhange et al. (2021) [22]	2	2	0	2	2	2	10	Pass
Ally and Balandya (2023) [23]	1	2	0	2	2	2	9	Pass
Bell et al. (2024) [24]	2	2	0	2	2	1	9	Pass
Onimoe and Rotz (2020) [25]	1	1	0	2	2	1	7	Fail
Ellis et al. (2016) [26]	2	2	0	1	1	1	7	Fail
Hoppe and Neumayr (2019) [27]	1	2	0	2	2	1	8	Fail

Two cross-sectional studies that met the JBI risk of bias criteria for good quality were included in our analysis [16], demonstrated in Table 5 below.

**Table 5:** The JBI critical appraisal checklist for cross-sectional studies used in our review We used yes, no, unclear, and not applicable. The passing score is >70%. We decided to include the author's manuscript to the editor [28] due to the fact that we had very few data on our research subject JBI: Joanna Briggs Institute Reference: [16]

	Crouch et al. (2018) [28]	Williams et al. (2021) [29]
Were the criteria for inclusion clearly defined?	Yes	Yes
Were the study subjects and the settings described in detail?	Yes	Yes
Was the exposure measured in a valid and reliable way?	No	Unclear
Were objective, standard criteria used for the measurement of the condition?	Yes	Yes
Were confounding factors identified?	Unclear	Yes
Were strategies to deal with confounding factors stated?	Unclear	Yes
Were the outcomes measured in a valid and reliable way?	Yes	Yes
Was appropriate statistical analysis used?	Yes	Yes
Score	<70%	>70%

Additionally, we used the NOS check tool [19] to evaluate one cohort research which met the standards as described in Table 6.

**Table 6:** The NOS assessment tool for the only cohort study in this review Results of NOS assessment tool for observation studies by review authors. The passing score is 7/9 N/A: not applicable; NOS: Newcastle-Ottawa Scale Reference: [19]

Author, date	Representativeness of the exposed cohort	Selection of the non-exposed cohort	Ascertainment of exposure	Demonstration that the outcome of interest was not present at the start of the study	Comparability of the cohort-based design/analysis	Assessment of the outcome	Was the follow-up long enough for the outcome to occur?	Adequacy of follow-up cohorts	Pass/fail
Nnajekwu et al. (2022) [30]	Yes	Yes	Yes	NA	Yes	Yes	Yes	Yes	Yes (included)

There were 373 men and women participants in this review among the eight articles that were chosen. The research was done in Jamaica, Nigeria, India, the United States, and Canada. Our review's findings demonstrated that individuals with SCD regularly receive folic acid, which raises their blood levels of folate. Table 7 below describes the articles retained in our review and their corresponding results.

**Table 7:** Selected articles included in this review SCA: sickle cell anemia; SCD: sickle cell disease; CI: confidence interval; RBC: red blood cell

Authors	Study type	Results
Ally and Balandya (2023) [23]	Narrative	Recommended folic acid supplementation in the management of SCD to prevent folate deficiency which could arise as a result of elevated RBC production.
Crouch et al. (2018) [28]	Cross-sectional	It was found that most SCD patients with leg ulcers were routinely taking folic acid. Folic acid is useful in wound healing among SCD patients.
Williams et al. (2021) [29]	Cross-sectional	Recommended prophylactic folic acid supplementation (1 mg/d) in all SCD patients. The analysis showed that individuals with both plasma and serum total folate results (n=8) showed a correlation coefficient (95% CI) of 0.984 (0.930, 0.996).
Dixit et al. (2018) [20]	Systematic review	There were significant differences between the folic acid and placebo groups with regard to serum folate values above 18 µg/l and values below 5 µg/l, respectively.
Bhange et al. (2021) [22]	Narrative	Recommended that every child with SCD should be given 1 mg of folic acid daily for life.
Hur and Durojaye (2023) [21]	Narrative	Patients with SCD who are on daily folic acid supplementation were found to have elevated folate levels.
Bell et al. (2024) [24]	Narrative	Demonstrated that folic acid supplementation may increase serum folate levels.
Nnajekwu et al. (2022) [30]	Cohort	Illustrated that during the crisis state of SCA, the median level of RBC folate was within normal value, while SCA children were on 5 mg folic acid daily.

 Discussion

Folic Acid Use and Dosage

According to our review, folic acid supplements ranging from 1 to 5 milligrams/day are typically used to avoid folate deficiency and the possibility of megaloblastic anemia in SCD patients. Folic acid is a water-soluble vitamin which is necessary for the activation of single-carbon molecules that produce purines, which are needed for the synthesis of polypeptides like DNA and RNA [31]. Folate is one of the most important nutrients for healthy cell division and RBC synthesis. It has been observed that the typical half-life of RBCs is 120-160 days in healthy persons, while it drops to between 15 and 20 days in SCD patients [13]. In addition, during sickle cell crises, the half-life is further reduced, which causes the bone marrow to become more active in order to replace the defective erythrocytes [32]. This alteration results in vitamin B9 breakage and increases the likelihood of folic acid insufficiency [14]. Based on these results, children and adults with SCA are routinely supplemented with folic acid every day to improve RBC production and prevent folate deficiency [20]. In most sub-Saharan African countries, the recommended daily dosage of folic acid among people living with SCD is 1-5 milligrams daily. The 5 milligrams of folic acid per day was reported in some reviewed articles by Ally and Balandya, Dixit et al., and Nnajekwu et al. [20,23,30]. Also, folic acid 1 milligram daily was reported in some reviewed articles by Williams et al., Bhange et al., and Hur and Durojaye [21,29,22]. However, it is not fully utilized in some nations, like the Democratic Republic of the Congo, where over 50% of patients were found not to be using folic acid [33].

Folate Supplementation and Folic Acid Deficiency Prevention

In order to prevent folic acid deficiency, which could result from increased RBC production, folic acid administration was advised in a 2023 study conducted by Ally and Balandya in the management of SCD [23,34]. There is conflicting evidence, according to Dixit et al., Williams et al., and Nnajekwu et al., that folic acid supplements are beneficial for SCD patients [20,29,30]. Folate supplementation is therefore recommended because macro- and micronutrient deficiencies are common in Africa and can contribute to as much as 45% of child mortality [35]. SCD is an inherited genetic disease with different complications such as leg ulcers. Patients with SCD may experience severe disruptions in their daily lives due to leg ulcers, and the prevalence of these lesions varies depending on factors such as socioeconomic status, geographic location, and genotype [36]. A healthy diet is essential for the healing of ulcers. Micronutrient deficiencies have been observed in SCD patients, and their daily dietary needs may be higher than those of healthy people [37]. The majority of SCD patients with leg ulcers were found to be taking folic acid on a regular basis in one of our review articles by Crouch et al. According to the report, the group with leg ulcers differed from the group without ulcers most significantly in terms of folic acid intake. It was found that SCD patients with the ulcer were taking folic acid twice as frequently more than the non-ulcer group. Vitamin B complex supplements contain nutrients like thiamine, pantothenic acid, and folic acid that aid in the healing of wounds [28,38]. Folate is one of the reactants that make S-adenosylmethionine (SAM), the universal methyl donor, and plays a role in one-carbon metabolism. Deficits in vitamins B2, B6, or B12 can modify methylation processes, reduce cellular proliferation, and impact vitamin B9-dependent nucleic acid synthesis, which can result in megaloblastic anemia [39]. All SCD patients should take a preventive folic acid supplement (1 mg/d) according to Williams et al. A correlation coefficient (95% CI) of 0.984 (0.930, 0.996) was found in their investigation for people with both plasma and serum total folate findings (n=8), indicating a very significant association between the two. The elevated levels of plasma/serum folate detected in non-fasting samples among certain participants in this investigation could perhaps be attributed to their recent intake of folic acid supplements. While vitamin B6 shortage was identified in certain participants, there was no evidence of vitamin B12 deficiency in this population [29]. Three commonly utilized prophylactic strategies for the treatment of SCD are penicillin, pneumococcal vaccination, and folate supplements [40]. Folic acid supplementation in individuals with SCD was investigated in the Dixit et al. study, a double-blind, placebo-controlled quasi-randomized trial. Over the course of a year, 117 children with homozygous SCD (SS) who were targeted to be between the ages of six months and four years participated in the study. Following trial entry at six and 12 months, blood folic acid titers for 80 out of 115 patients (about 70%) were collected. The results indicated that serum folate levels above 18 µg/l and below 5 µg/l, respectively, differed significantly between the folic acid and placebo groups. Values exceeding 18 µg/l were found in 33 out of 41 (81%) participants in the folic acid group, while only six out of 39 (15%) people in the placebo (calcium lactate) group showed similar results. Furthermore, no participants in the folic acid group had serum folate levels below 5 µg/l, whereas 15 of 39 (39%) participants in the placebo group did. These results were consistent with earlier research by Ally and Balandya [20,23]. Furthermore, it was observed that, at baseline and one year later, there were no appreciable variations in the trial groups' total Hb concentrations. Side effects of folic acid supplementation like priapism, the risk of masking cobalamin deficiency neuropsychiatric manifestation, and other clinical manifestations of acute splenic sequestration were not assessed. Nevertheless, the investigation showed that the placebo group experienced more recurrent bouts of dactylitis, with two or more attacks occurring in 10 out of 56 people, as opposed to two out of 59 in the folic acid group (P<0.05) [20]. A comprehensive update review on the current management of SCA by Bhange et al. recommended that every child with SCD should be given 1 mg of folic acid daily for life [22].

Folic Acid Supplementation and Increase in Folate Serum Levels

According to research conducted by Hur and Durojaye, patients with SCD who take folic acid supplements on a daily basis had enhanced levels of folate. These findings are consistent with those reported by Williams et al. [21,29]. Sickle cell crises are characterized by acute episodes that aggravate clinical manifestations of SCA, such as pain, jaundice, or anemia. During sickle cell crises, the half-life of RBCs is further reduced, leading to increased bone marrow activity to replace the defective erythrocytes [40]. A study by Nnajekwu et al. showed that while SCA patients were taking a daily 5 mg supplement of folic acid, the median level of RBC folate remained within normal limits during the crisis state. The results matched those of Dixit et al. [20]. Even though hemolysis, a symptom of sickle cell crisis, may raise the need for folate to replace the damaged cells, the SCA children's normal mean red cell folate levels may be explained by their use of vitamin B9 supplements. Furthermore, when compared to crisis situations, the majority of SCA youngsters had noticeably greater folate levels during steady state. Nonetheless, during the SCA crisis, 8% of the children with SCA had a folate shortage. Compared to a vaso-occlusive crisis, the likelihood of having a folic acid deficiency was eight times higher in anemic crises. This is different from the findings of the research that Williams et al. undertook. The significant hemolysis associated with anemic crises may account for the higher number of people reported to be vitamin B9 deficient during this period. This alteration increases the need for folic acid in the synthesis of DNA and the creation of new RBCs to replace the ones that are destroyed. The danger of folate insufficiency may rise if the stocks of folate are depleted. The existence of a folate shortage during an anemic crisis despite folic acid supplementation implies that the dose of folic acid may need to be adjusted to ensure a steady supply during the crisis [14,29,30]. A study on new treatment and challenging nutritional intervention for SCD patients by Bell et al. demonstrated that vitamin B9 supplementation may increase serum folate levels, but the effect of folic acid on SCA still needs better clarification. These individuals may be more susceptible to severe painful crises, inflammation, opportunistic infections, and stunting if they have micronutrient deficits. Iron, zinc, copper, folic acid, pyridoxine, and vitamin E are some of these micronutrients [24,41]. Most SCD patients were taking folic acid supplements and had normal or raised blood folate levels, according to the results of the publications we chose for our study. However, little is known about how folic acid helps with SCD. A few limitations of our review included the following: a small sample size, studies were only included because they were conducted in a particular region, and the majority of the studies that were included did not examine the effects of folic acid on clinical outcomes in SCD patients.

## Conclusions

SCD is a non-communicable and congenital blood disorder. It consists of a set of clinical disorders that impact Hb because of an aberrant polymerized oxygen-deprived Hb genetic coding. Due to the fact that SCD patients produce more RBCs than healthy individuals, nearly all SCD patients receive daily folic acid supplements. On the other hand, not enough information is available on folic acid's potential benefits in the management of SCD. Based on the review, a larger clinical trial is required to establish the effectiveness of folic acid in the management of SCD.
